# Supplementary material for: HIVprotI: an integrated web based platform for prediction and design of HIV proteins inhibitors
Source: J Cheminform. 2018 Mar 9;10:12. doi: 10.1186/s13321-018-0266-y (PMC5845081; doi:10.1186/s13321-018-0266-y)
Supplement: Supplementary file 2 — Additional file 2. Source code of HIVProtI web server. [file 13321_2018_266_MOESM2_ESM.zip › HIVprotI_Source-code/hivprotI/weka-3-6-6/doc/weka/core/SparseInstance.html]

SparseInstance


---


|  |  |  |
| --- | --- | --- |
| **Overview** \| **Package** \| **Class** \| **Tree** \| **Deprecated** \| **Index** \| **Help** \| **Weka's home** | |  |
| **PREV CLASS**   **NEXT CLASS** | **FRAMES**    **NO FRAMES**     **All Classes** |  |
| SUMMARY: NESTED \| FIELD \| CONSTR \| METHOD | DETAIL: FIELD \| CONSTR \| METHOD |  |


---


## weka.core Class SparseInstance

```
java.lang.Object
  weka.core.Instance
      weka.core.SparseInstance
```

**All Implemented Interfaces:**: java.io.Serializable, Copyable, RevisionHandler

**Direct Known Subclasses:**: BinarySparseInstance

---

``` public class SparseInstance extends Instance ```

Class for storing an instance as a sparse vector. A sparse instance
only requires storage for those attribute values that are non-zero.
Since the objective is to reduce storage requirements for datasets
with large numbers of default values, this also includes nominal
attributes -- the first nominal value (i.e. that which has index 0)
will not require explicit storage, so rearrange your nominal attribute
value orderings if necessary. Missing values will be stored
explicitly.

**Version:**
:   $Revision: 5970 $

**Author:**
:   Eibe Frank

**See Also:**: Serialized Form

---

**Constructor Summary**

`SparseInstance(double weight,
double[] attValues)`
  
          Constructor that generates a sparse instance from the given
parameters.

`SparseInstance(double weight,
double[] attValues,
int[] indices,
int maxNumValues)`
  
          Constructor that inititalizes instance variable with given
values.

`SparseInstance(Instance instance)`
  
          Constructor that generates a sparse instance from the given
instance.

`SparseInstance(int numAttributes)`
  
          Constructor of an instance that sets weight to one, all values to
be missing, and the reference to the dataset to null.

`SparseInstance(SparseInstance instance)`
  
          Constructor that copies the info from the given instance.


| **Method Summary** | |
| --- | --- |
| `Attribute` | `attributeSparse(int indexOfIndex)`             Returns the attribute associated with the internal index. |
| `java.lang.Object` | `copy()`             Produces a shallow copy of this instance. |
| `java.lang.String` | `getRevision()`             Returns the revision string. |
| `int` | `index(int position)`             Returns the index of the attribute stored at the given position. |
| `boolean` | `isMissing(int attIndex)`             Tests if a specific value is "missing". |
| `int` | `locateIndex(int index)`             Locates the greatest index that is not greater than the given index. |
| `static void` | `main(java.lang.String[] options)`             Main method for testing this class. |
| `Instance` | `mergeInstance(Instance inst)`             Merges this instance with the given instance and returns the result. |
| `int` | `numAttributes()`             Returns the number of attributes. |
| `int` | `numValues()`             Returns the number of values in the sparse vector. |
| `void` | `replaceMissingValues(double[] array)`             Replaces all missing values in the instance with the values contained in the given array. |
| `void` | `setValue(int attIndex, double value)`             Sets a specific value in the instance to the given value (internal floating-point format). |
| `void` | `setValueSparse(int indexOfIndex, double value)`             Sets a specific value in the instance to the given value (internal floating-point format). |
| `double[]` | `toDoubleArray()`             Returns the values of each attribute as an array of doubles. |
| `java.lang.String` | `toString()`             Returns the description of one instance in sparse format. |
| `double` | `value(int attIndex)`             Returns an instance's attribute value in internal format. |

**Methods inherited from class weka.core.Instance**

`attribute, classAttribute, classIndex, classIsMissing, classValue, dataset, deleteAttributeAt, enumerateAttributes, equalHeaders, hasMissingValue, insertAttributeAt, isMissing, isMissingSparse, isMissingValue, missingValue, numClasses, relationalValue, relationalValue, setClassMissing, setClassValue, setClassValue, setDataset, setMissing, setMissing, setValue, setValue, setValue, setWeight, stringValue, stringValue, toString, toString, value, valueSparse, weight`

**Methods inherited from class java.lang.Object**

`equals, getClass, hashCode, notify, notifyAll, wait, wait, wait`

**Constructor Detail**

### SparseInstance

```
public SparseInstance(Instance instance)
```

:   Constructor that generates a sparse instance from the given
    instance. Reference to the dataset is set to null.
    (ie. the instance doesn't have access to information about the
    attribute types)

    **Parameters:**: `instance` - the instance from which the attribute values and the weight are to be copied

---


### SparseInstance

```
public SparseInstance(SparseInstance instance)
```

:   Constructor that copies the info from the given instance.
    Reference to the dataset is set to null.
    (ie. the instance doesn't have access to information about the
    attribute types)

    **Parameters:**: `instance` - the instance from which the attribute info is to be copied

---


### SparseInstance

```
public SparseInstance(double weight,
                      double[] attValues)
```

:   Constructor that generates a sparse instance from the given
    parameters. Reference to the dataset is set to null.
    (ie. the instance doesn't have access to information about the
    attribute types)

    **Parameters:**: `weight` - the instance's weight: `attValues` - a vector of attribute values

---


### SparseInstance

```
public SparseInstance(double weight,
                      double[] attValues,
                      int[] indices,
                      int maxNumValues)
```

:   Constructor that inititalizes instance variable with given
    values. Reference to the dataset is set to null. (ie. the instance
    doesn't have access to information about the attribute types)
    Note that the indices need to be sorted in ascending order. Otherwise
    things won't work properly.

    **Parameters:**: `weight` - the instance's weight: `attValues` - a vector of attribute values (just the ones to be stored): `indices` - the indices of the given values in the full vector (need to be sorted in ascending order): `maxNumValues` - the maximium number of values that can be stored

---


### SparseInstance

```
public SparseInstance(int numAttributes)
```

:   Constructor of an instance that sets weight to one, all values to
    be missing, and the reference to the dataset to null. (ie. the instance
    doesn't have access to information about the attribute types)

    **Parameters:**: `numAttributes` - the size of the instance


**Method Detail**

### attributeSparse

```
public Attribute attributeSparse(int indexOfIndex)
```

:   Returns the attribute associated with the internal index.

    :   **Overrides:**: `attributeSparse` in class `Instance`
    :   **Parameters:**: `indexOfIndex` - the index of the attribute's index **Returns:**: the attribute at the given position **Throws:**: `UnassignedDatasetException` - if instance doesn't have access to a dataset

---


### copy

```
public java.lang.Object copy()
```

:   Produces a shallow copy of this instance. The copy has
    access to the same dataset. (if you want to make a copy
    that doesn't have access to the dataset, use
    `new SparseInstance(instance)`

    :   **Specified by:**: `copy` in interface `Copyable` **Overrides:**: `copy` in class `Instance`
    :   **Returns:**: the shallow copy

---


### index

```
public int index(int position)
```

:   Returns the index of the attribute stored at the given position.

    :   **Overrides:**: `index` in class `Instance`
    :   **Parameters:**: `position` - the position **Returns:**: the index of the attribute stored at the given position

---


### isMissing

```
public boolean isMissing(int attIndex)
```

:   Tests if a specific value is "missing".

    :   **Overrides:**: `isMissing` in class `Instance`
    :   **Parameters:**: `attIndex` - the attribute's index **Returns:**: true if the value is "missing"

---


### locateIndex

```
public int locateIndex(int index)
```

:   Locates the greatest index that is not greater than the
    given index.

    :   **Returns:**: the internal index of the attribute index. Returns -1 if no index with this property could be found

---


### mergeInstance

```
public Instance mergeInstance(Instance inst)
```

:   Merges this instance with the given instance and returns
    the result. Dataset is set to null.

    :   **Overrides:**: `mergeInstance` in class `Instance`
    :   **Parameters:**: `inst` - the instance to be merged with this one **Returns:**: the merged instances

---


### numAttributes

```
public int numAttributes()
```

:   Returns the number of attributes.

    :   **Overrides:**: `numAttributes` in class `Instance`
    :   **Returns:**: the number of attributes as an integer

---


### numValues

```
public int numValues()
```

:   Returns the number of values in the sparse vector.

    :   **Overrides:**: `numValues` in class `Instance`
    :   **Returns:**: the number of values

---


### replaceMissingValues

```
public void replaceMissingValues(double[] array)
```

:   Replaces all missing values in the instance with the
    values contained in the given array. A deep copy of
    the vector of attribute values is performed before the
    values are replaced.

    :   **Overrides:**: `replaceMissingValues` in class `Instance`
    :   **Parameters:**: `array` - containing the means and modes **Throws:**: `java.lang.IllegalArgumentException` - if numbers of attributes are unequal

---


### setValue

```
public void setValue(int attIndex,
                     double value)
```

:   Sets a specific value in the instance to the given value
    (internal floating-point format). Performs a deep copy
    of the vector of attribute values before the value is set.

    :   **Overrides:**: `setValue` in class `Instance`
    :   **Parameters:**: `attIndex` - the attribute's index: `value` - the new attribute value (If the corresponding attribute is nominal (or a string) then this is the new value's index as a double).

---


### setValueSparse

```
public void setValueSparse(int indexOfIndex,
                           double value)
```

:   Sets a specific value in the instance to the given value
    (internal floating-point format). Performs a deep copy
    of the vector of attribute values before the value is set.

    :   **Overrides:**: `setValueSparse` in class `Instance`
    :   **Parameters:**: `indexOfIndex` - the index of the attribute's index: `value` - the new attribute value (If the corresponding attribute is nominal (or a string) then this is the new value's index as a double).

---


### toDoubleArray

```
public double[] toDoubleArray()
```

:   Returns the values of each attribute as an array of doubles.

    :   **Overrides:**: `toDoubleArray` in class `Instance`
    :   **Returns:**: an array containing all the instance attribute values

---


### toString

```
public java.lang.String toString()
```

:   Returns the description of one instance in sparse format.
    If the instance doesn't have access to a dataset, it returns the
    internal floating-point values. Quotes string values that contain
    whitespace characters.

    :   **Overrides:**: `toString` in class `Instance`
    :   **Returns:**: the instance's description as a string

---


### value

```
public double value(int attIndex)
```

:   Returns an instance's attribute value in internal format.

    :   **Overrides:**: `value` in class `Instance`
    :   **Parameters:**: `attIndex` - the attribute's index **Returns:**: the specified value as a double (If the corresponding attribute is nominal (or a string) then it returns the value's index as a double).

---


### main

```
public static void main(java.lang.String[] options)
```

:   Main method for testing this class.

---


### getRevision

```
public java.lang.String getRevision()
```

:   Returns the revision string.

    :   **Specified by:**: `getRevision` in interface `RevisionHandler` **Overrides:**: `getRevision` in class `Instance`
    :   **Returns:**: the revision


---


|  |  |  |
| --- | --- | --- |
| **Overview** \| **Package** \| **Class** \| **Tree** \| **Deprecated** \| **Index** \| **Help** \| **Weka's home** | |  |
| **PREV CLASS**   **NEXT CLASS** | **FRAMES**    **NO FRAMES**     **All Classes** |  |
| SUMMARY: NESTED \| FIELD \| CONSTR \| METHOD | DETAIL: FIELD \| CONSTR \| METHOD |  |


---
